# Superior efficacy of rituximab-based chemoimmunotherapy as an initial therapy in newly diagnosed patients with B cell indolent lymphomas: long-term results from a single center in China

**DOI:** 10.1186/s12885-015-1534-0

**Published:** 2015-07-29

**Authors:** Zengjun Li, Fei Li, Shuhua Yi, Zhimin Gu, Zhen Yu, Yan Xu, Xiaoyan Feng, Wei Liu, Dehui Zou, Junyuan Qi, Fenghuang Zhan, Lugui Qiu

**Affiliations:** 1 State Key Laboratory of Experimental Hematology, Institute of Hematology and Blood Disease Hospital, Chinese Academy of Medical Sciences and Peking Union Medical College, 288 Nanjing Road, Heping District, Tianjin, 300020 China; 2 Department of Hematology, The First Affiliated Hospital of Nanchang University, NanChang, 330006 China; 3 Umbilical Cord Blood Bank of Tianjin, Tianjin, 300020 China; 4 Department of Internal Medicine, University of Iowa Carver College of Medicine, Iowa City, IA 52246 USA

**Keywords:** B cell indolent lymphoma, Chronic lymphocytic leukemia, Rituximab, Chemoimmunotherapy, Prognosis

## Abstract

**Background:**

Rituximab has been confirmed to improve the survival of patients with B cell indolent non-Hodgkin lymphomas (B-iNHLs) in Western world as previously reported, however, it is rarely reported in Chinese cohort. This study is to investigate the efficacy and safety of rituximab-based chemoimmunotherapy and select subpopulations most sensitive to the regimen in Chinese B-iNHL patients.

**Methods:**

334 B-iNHL patients from our center were retrospectively assessed.

**Results:**

Patients received R-based chemoimmunotherapy showed significantly higher rates of overall response (OR) (93.0 % *vs.* 53.4 %, *P* < 0.001) and complete response (CR) (63.3 % *vs.* 16.0 %, *P* < 0.001) compared with the patients received other therapies. Survival analysis showed that rituximab-based chemoimmunotherapy could obviously improve the progression-free survival (PFS) (110 *vs.* 49 months, *P* = 0.001) and overall survival (OS) (120 *vs.* 72 months, *P* < 0.001) in patients with B-iNHLs. Interestingly, in chronic lymphocytic leukemia (CLL) patients, we found that the patients with β2-microglobulin (β2-MG) < 3.5 mg/L, lactate dehydrogenase (LDH) < 220 U/L, zeta-chain-associated protein kinase 70 (ZAP-70) negative, and non high-risk genetic abnormality could achieve more benefits from R-based regimens with higher CR rate (*P* = 0.003, 0.029, 0.013 and 0.038, respectively). Meanwhile, more CLL patients achieved minimal residual disease (MRD) negative after rituximab-based treatment (46.5 % *vs.* 10.3 %, *P* < 0.001). Moreover, CLL patients with MRD < 1 %, LDH < 220 U/L, complete remission (CR) or partial remission (PR), β2-MG < 3.5 mg/L and non high-risk cytogenetic abnormality showed superior outcome compared to the controls (*P* = 0.001, 0.000, 0.000, 0.001 and 0.013, respectively). No other side-effects increased in chemoimmunotherapy group except the elevation of grade 3–4 neutropenia.

**Conclusions:**

Our results demonstrate the superior efficacy of rituximab–based chemoimmunotherapy as an initial therapy in Chinese cohort with newly diagnosed B-iNHLs and further identify subpopulations that are more sensitive to R-based chemoimmunotherapy in CLL group.

## Background

B cell indolent non-Hodgkin lymphomas (B-iNHLs) are lymphoid neoplasms that are characterized by abnormal proliferation of monoclonal mature B lymphocytes in peripheral blood, bone marrow, spleen or lymph nodes. Different entities are separated by clinical and histopathological features according to the classification of World Health Organization (WHO), mainly including chronic lymphocytic leukemia (CLL), follicular lymphoma (FL), nodal marginal zone lymphoma (NMZL), splenic B-cell marginal zone lymphoma (SMZL), lymphoplasmacytoid lymphoma/Wadenström macroglobulinemia (LPL/WM), hairy cell leukemia (HCL), and chronic B lymphoproliferative disease undefined (BLPD-U). These disorders are frequently grouped together under the category of “B-chronic lymphoproliferative disorders, BLPD”. B-iNHLs are still considered as incurable diseases [1, 2], except the treatment with allogeneic hematopoietic stem-cell transplantation (Allo-HSCT) that is considered as an appropriate therapy for selected patients with poor prognosis. However, remarkable progress has been achieved in B-cell lymphomas over the past 2–3 decades. Highly active treatment reagents and combinations such as the purine analog fludarabine as well as rituximab-based regimens result in high and durable response rate [3–7].

Rituximab (R) is a chimeric human-mouse monoclonal antibody that targets the CD20 antigen that is commonly expressed on B lymphocytes, but not on plasma cells or hematopoietic stem cells. Rituximab has achieved some exciting results during the last decade through increasing chemosensitivity and consolidating treatment responses in B cell lymphomas [5–9]. Rituximab binds to CD20 on the surface of B cells, which results in rapid and durable depletion of normal and malignant B cells via multiple mechanisms including antibody-dependent cell-mediated cytotoxicity (ADCC), complement-dependent cytotoxicity (CDC) and direct induction of apoptosis [10]. A combination of rituximab and cytostatic drugs has now become standard first-line therapy for some indolent B cell lymphomas [11, 12].

Due to the low incidence of indolent B cell lymphomas in China, the efficacy and safety of rituximab-based chemoimmunotherapy were rarely reported in Chinese patients. In order to determine patient’s pretreatment characteristics associated with superior outcomes and identify untreated patients most appropriate for the initial regimens in Chinese cohort with newly diagnosed indolent B cell lymphomas, we retrospectively analyzed the clinical therapy response, survival and safety of rituximab-based regimen as an initial therapy in our center since 1999.

## Methods

### Ethics statement

This study was approved by the ethic committee of the Institute of Hematology, Chinese Academy of Medical Sciences, and Peking Union Medical College, according to the guidelines of the 1996 Helsinki Declaration (reference number: NI2015003-EC-1). Written informed consent was obtained from all patients.

### Patients

A total of 695 patients with indolent B cell lymphomas were admitted to the lymphoma center of Blood Disease Hospital of Chinese Academy of Medical Sciences in Tianjin, China. Diagnosis was determined according to the 2008 World Health Organization classification. BLPD-U was diagnosed when patients could not be classified as a definite type by pathology immunohistochemistry, immunophenotype or cytogenetic analysis. Patients who were pretreated with a purine analogue or other chemotherapy, subsequently received rituximab-based chemoimmunotherapy when relapsed; The patients who needed “watch and wait” or were lost to follow-up were excluded from this study. Finally, 334 evaluable indolent B cell lymphomas patients with an indication for treatment and complete clinical data were included in this study. All patients were staged according to the Ann Arbor or Rai system and matched treatment indications appropriate for different B lymphomas [1, 13–15].

Pretreatment evaluation was consisted of a history and physical examination, laboratory tests including peripheral blood examination, renal and liver function, lactic dehydrogenase level (LDH), serum beta2-microglobulin (β2-MG), C-reactive protein (CRP), serum immunoglobulin levels, hemolysis and virus inspection. Patients underwent bone marrow aspiration for the analysis of immunophenotyping and metaphase karyotype, bone marrow biopsy, and CT scans of the chest, abdomen, and pelvis. Patients were routinely detected cytogenetic abnormalities including IgH, p53, RB-1, ATM by fluorescence in situ hybridization (FISH) since 2006.

### Treatment regimens

Patients reveived rituximab-based chemoimmunotherapy (R-CHOP-like [rituximab, cyclophosphamide, doxorubicin, vincristine, and prednisone], R-FC [rituximab, fludarabine, cyclophosphamide], or other R-based regimens) and other non-R-based therapies including chlorambucil, CHOP-Like, or FC. Rituximab was administered on day 0 at 375 mg/m^2^ for cycle 1 and 500 mg/m^2^ for all subsequent cycles in CLL patients and 375 mg/m^2^ on day 0 every cycle in patients with other B cell lymphomas. Dexamethasone (10 mg) and promethazine hydrochloride (25 mg) were administered before rituximab for each course. The treatments including hydration, alkalization and protection of liver, heart, stomach were routinely given. Myeloid growth factors were not routinely administered only if patients experienced grade 3 or 4 neutropenia. Red blood cells or platelets (PLT) suspension was infused when hemoglobin (Hb) < 70 g/L or PLT < 20 × 10^9^/L. Courses were repeated every four weeks depending on the recovery of neutrophil or platelet counts. Dose reductions for chemotherapy, but not rituximab, were made if patients experienced prolonged grade 3 or 4 hematologic toxicity or infections. The therapy effects were evaluated every two courses. Patients in R-group who achieved stable treatment response (≥ partial response, PR) were eligible to receive maintenance therapy up to 12–14 cycles of rituximab (375 mg⁄m^2^) every three months until relapse or progression for a maximum of two years. Other patients in non-R-group received “watch and wait” or maintenance therapy with chlorambucil or interferon until relapse or progression after achieving stable treatment response.

### Response criteria

Treatment response was evaluated at least two months after completion of therapy. Responses to treatment, which is divided into complete remission (CR), PR, stable disease (SD) and progression disease (PD) were determined according to standard criteria [1, 13–16]. CR was defined as the complete disappearance of all detectable sites and symptoms of disease. PR was defined as 50 % or greater improvement in the disease localization. PD was defined as a greater than 25 % increase in a size of previously documented disease or the appearance of disease at any site or shift to a more aggressive histological pattern. SD was defined as not in keeping with the criteria of CR, PR and PD. Overall response rate (ORR) was defined as CR plus PR.

Flow cytometry evaluation of bone marrow aspirate was performed to estimate minimal residual disease (MRD) by evaluating CD5^+^/CD19^+^ lymphocytes in CLL patients. MRD testing was performed before the initial therapy, every two cycles of therapy, two months after the last treatment cycle, subsequently every three months. MRD was considered as negativity by four-color flow cytometry at least twice less than 10^−4^ of CD5^+^/CD19^+^ coexpressing cells.

Severity and frequency of side effects were graded according to the Common Toxicity Criteria (CTC) Version 4.0 of the National Cancer Institute.

### Statistical analysis

Progression-free survival (PFS) was calculated from the date of treatment initiation until disease progression or death, and overall survival (OS) was calculated from the date of treatment initiation to death. Survival curves were graphed by the Kaplan–Meier method, differences between curves were analyzed for statistical significance using the log-rank test. Categorical variables were compared using nonparametric tests and the Pearson’s Chi-square test. Multivariate analysis was performed using the cox-regression method. A *P* value of <0.05 was considered statistically significant. All data analyses were performed using the statistical software SPSS version 20.0.

## Results

### Patients’ characteristics

In all 334 evaluable patients, there were 151 CLL, 24 MZL (13 SMZL and 11 NMZL), 17 HCL, 36 LPL/WM, 41 FL and 65 BLPD-U. 128 patients received rituximab-based chemoimmunotherapy, while 206 patients received non-rituximab-based therapy as initial therapies. The baseline characteristics of patients were shown in Table 1. The median age of 334 patients was 56 years old (range, 19–87 yr). The parameters such as age, sex, performance status (Eastern cooperative Oncology Group score, ECOG score), serum levels of β2-MG, genomic aberrations were well balanced except that the median level of leukocyte count was higher in chemotherapy group (*P* = 0.021) and the percentages of CD20^+^, ZAP-70^+^ ( zeta-chain-associated protein kinase 70 positive) and CD38^+^ cells were higher in chemoimmunotherapy group (*P* = 0.001, 0.027 and 0.010) (Table 1).

**Table 1:** Clinical features of 334 patients with B-iNHLs

Characteristics	Chemotherapy	Chemoimmunotherapy
Number of patients	206	128
Median Age, y (Range)	59 (26–87)	54 (19–82)
≤60, %	108 (52.4)	89 (69.5)
60-70	72 (35.0)	28 (21.9)
≥70	26 (12.6)	11 (8.6)
Male/Female, %	71/29	65.4/34.6
ECOG performance status, %		
0	49.4	52.3
1-2	45.9	44.7
3	4.7	3.0
Leukocyte count, 10^9^/L (range)	18.0 (83–382)	11.5 (63–300)^1^*
Hemoglobin, g/L (range)	113 (21–169)	112 (41–177)
Thrombocyte count, 10^9^/L (range)	120 (4–759)	136 (10–577)
β2-microglobulin, mg/L (range)	3.69 (1–14.5)	3.41 (1–13.4)
LDH, U/L (range)	166 (16–977)	164 (17–1892)
CD20^+^ cells by flow cytometry, %	143/178 (80.3 %)	99/105 (94.3 %)^2^*
Cytogenetic abnormalities, %		
Del (13q)	15/99 (15.2 %)	11/100 (11.0 %)
Del (17p)	13/91 (14.3 %)	13/87 (14.9 %)
IgH	27/99 (27.3 %)	39/102 (38.2 %)
ZAP70 positive	19/61 (31.1 %)	18/33 (54.5 %)^3^*
CD38 positive	38/146 (26.0 %)	36/85 (42.4 %)^4^*
Histology ( N)		
CLL (151)	107	44
FL (41)	13	28
NMZL (11)	5	6
SMZL (13)	7	6
HCL (17)	14	3
LPL (36)	22	14
BLPD-U (65)	38	27

*CLL* Chronic lymphocytic leukemia, *FL* Follicular lymphoma, *NMZL* Nodal marginal zone lymphoma, *SMZL* Splenic B-cell marginal zone lymphoma, *LPL* Lymphoplasmacytoid lymphoma, *BLPD-U* B lymphoproliferative disease–unclassified

^1^*P = 0.021; ^2^* *P* = 0.001; ^3^*P = 0.027; ^4^*P = 0.010

### Response to treatment

Except seven patients received less than four courses of R-based treatment due to poor treatment response, all of other patients in R group received more than four courses of R-based therapy. The median treatment course of chemoimmunotherapy was 6.0 (range, 2–12 regimens). The rates of ORR (93.0 % *vs.* 53.4 %, *P* < 0.001) and CR (63.3 % *vs.* 16.0 %, *P* < 0.001) were much superior in patients with R-based chemoimmunotherapy than the patients with other therapies. The rates of treatment response in different BLPD subgroups were shown in Table 2. Nine patients were not responsive to R-based treatment, seven patients had SD and the other two had PD. The characteristics of nine patients with no response to R-based treatment were shown in Table 3.

**Table 2:** The rates of treatment response in different BLPD subgroups

Subgroup (NO.)	CR (n, %)	*P* value	ORR (n, %)	*P* value
CLL (151)		<0.001		<0.001
R-group (44)	24 (54.5 %)		40 (90.9 %)	
Non-R-group (107)	14 (13.1 %)		48 (44.9 %)	
FL (41)		0.044		0.008
R-group (28)	20 (71.4 %)		28 (100 %)	
Non-R-group (13)	5 (38.5 %)		10 (76.9 %)	
Other BLPD (142)		<0.001		<0.001
R-group (56)	37 (66.1 %)		51 (91.1 %)	
Non-R-group (86)	14 (16.3 %)		52 (60.5 %)

**Table 3:** The characteristics of nine patients with no response to R-based treatment (*N* = 9)

No of patients	Case 1	2	3	4	5	6	7	8	9
Disease	CLL	CLL	CLL	CLL	LPL	MZL	BLPD-U	BLPD-U	BLPD-U
Age (years old)	70	52	46	69	76	64	70	76	82
Sex (F/M)	F	F	F	M	M	F	M	M	M
ECOG score	0	0	1	0	0	0	1	0	1
β2-MG (mg/ L)	4.67	1.96	-	4.2	3.78	2.08	2.63	6.93	-
LDH (U/L)	228	224	1892	155	221	159	198	232	-
Del (13q)	-	-	-	-	-	-	-	-	-
Del (17p)	-	+	-	-	-	-	^a^	^a^	-
IgH	-	+	+	+	-	-	-	-	-
Del (ATM)	+	-	-	-	-	-	-	-	-
Complex karyotype	no	no	yes	no	no	no	yes	no	no
ZAP70	-	+	-	-	-	^a^	^a^	^a^	-
CD38	-	+	-	+	+	+	-	-	-
Courses of R-regimens	2	3	8	2	4	2	2	3	2
Treatment response	SD	PD	SD	SD	SD	PD	SD	SD	SD
PFS (months)	4	4	9	25	5	10	3.5	8	30
OS (months)	6	44	12	30	5	10	3.5	10	33
Outcome	dead	alive	dead	dead	dead	dead	dead	dead	dead

*β2-MG* β2-microglobulin, *LDH* lactic dehydrogenase level, *Complex karyotype* the chromosomal aberrations involving more than two chromosomes or three or more cleavage sites

^a^undetected

### CLL group

44 CLL patients received R-based chemoimmunotherapy, 90.9 % of patients revealed the response (CR + PR) to R-based chemoimmunotherapy and 54.5 % of patients achieved CR. In comparison, only 44.9 % of patients responded and 13.1 % of patients achieved CR (*P* < 0.001) in 107 CLL patients received chemotherapy.

### FL group

In R-group, 100 % (28/28) of patients achieved treatment response and 71.4 % of patients achieved CR, which was superior to the patients in the chemotherapy group (*P* = 0.008 and 0.044, respectively).

### Other BLPD group

Due to limited number of other BLPD patients including MZL, HCL, LPL/WM and BLPD-U, we classified these patients as one group. Similarly, The R group also showed more promising outcome than the non-R-group (ORR: 91.1 % *vs.* 60.5 %, *P* < 0.001; CR: 66.1 % *vs.* 16.3 %, *P* < 0.001). Subgroup analysis showed that the patients with R-based regimens also achieved higher rate of CR than the patients in non-R group (64.3 % *vs.*9.1 %, *P* < 0.001) in 36 patients with LPL/WM. However, no different rates of CR and ORR in 24 MZL patients between the R and non-R groups were observed (CR: 66.7 % *vs.* 33.3 %, *P* = 0.102; ORR: 91.7 % *vs.* 75.0 %, *P* = 0.273). Three HCL patients received rituximab-based initial treatment for severe skin infection and splenic infarction. One patient receiving 5 courses of RFC achieved CR while the other two patients receiving 4 courses of R-COP (rituximab combined with vincristine, cyclophosphamide and prednisone) achieved PR (ORR: 100 %). Among other fourteen patients receiving the treatments including interferon, fludarabine or chlorambucil, one patient achieved CR and nine patients achieved PR (ORR: 71.4 %).

### Correlation of clinical parameters and achieving CR in patients with R-based treatment

To determine the pretreatment characteristics associated with CR, we divided 128 patients with R-based chemoimmunotherapy into three subgroups: CLL, FL and other BLPD. In CLL subgroup, we further found patients with β2-MG < 3.5 mg/L, LDH < 220 U/L, ZAP-70 negative, and non high risk genetic abnormality benefited more from the R-based regimens with higher CR rate than control patients (*P* = 0.003, 0.029, 0.013 and 0.038) (Table 4). Moreover, in FL group, patients with low or medium risk FL International Prognostic Index (FLIPI) score had higher CR rate after receiving R-based treatments (*P* = 0.020) (Table 5). Due to the diversity of clinical and biological features in other BLPD patients, we didn’t find the specific pretreatment characteristics associated with CR when various clinical factors including Hb, PLT, age, β2-MG, LDH, albumin (ALB), Ann Arbor stage III-IV and bone marrow infiltration were analyzed.

**Table 4:** Correlation of clinical parameters and achieving CR in the CLL patients receiving R-based immunochemotherapy (*n* = 44)

Characteristic	Number	CR (n, %)	*P*
Age (years)			0.263
< 60	26	16 (61.5)	
≥ 60	18	8 (44.4)	
Rai risk stratification			0.405
Low or medium risk	25	15 (60.0)	
High risk	19	9 (47.4)	
β2-MG (mg/L)			0.003
< 3.5	15	12 (80.0)	
≥ 3.5	18	5 (27.8)	
LDH (U/L)			0.029
< 220	30	20 (66.7)	
≥ 220	13	4 (30.8)	
ZAP-70			0.013
Positive	15	4 (26.7)	
Negative	11	8 (72.7)	
CD38			0.478
Positive	11	5 (45.5)	
Negative	24	14 (58.3)	
Genetic abnormalities			0.038
High risk	15	5 (33.3)	
Non high risk	27	18 (66.7)	

High risk genetic abnormalities: del (p53) or del (ATM) or complex karyotype

**Table 5:** Correlation of clinical parameters and achieving CR in the FL patients receiving R-based immunochemotherapy (*n* = 28)

Characteristic	Number	CR (n, %)	*P*
Age(years)			0.486
< 60	26	19 (73.1)	
≥ 60	2	1 (50.0)	
FLIPI			0.020
Low or medium risk (≤2)	22	18 (81.8)	
High risk (≥3)	6	2 (33.3)	
Bone marrow involvement			
Yes	19	12 (63.2)	0.159
no	9	8 (88.9)	
β2-MG (mg/L)			0.967
< 3.5	17	12 (70.6)	
≥ 3.5	7	5 (71.4)	
LDH (U/L)			0.334
< 220	21	16 (76.2)	
≥ 220	7	4 (57.1)	

### MRD assessment

MRD was assessed in 150 evaluable CLL patients. 46.5 % (20/43) of patients achieved MRD negative during or after the treatment cycles in R-based group. However, in chemotherapy group, only 10.3 % of patients (11/107) achieved MRD negative (46.5 % *vs.* 10.3 %, *P* < 0.001). It suggested more CLL patients achieved MRD negative via R-based chemoimmunotherapy. Due to the limited number of patients in other groups, we did not observe the significant difference of MRD between R and non-R groups.

### Survival analysis

**Fig. 1:**
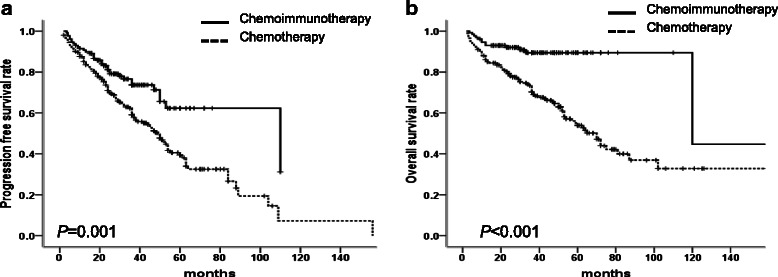
The comparison of outcomes between R-based chemoimmunotherapy and chemotherapy groups in 334 B-iNHLs patients. **a** Patients with R-based chemoimmunotherapy had superior PFS than patients with chemotherapy (110 *vs.* 49 months, *P* = 0.001). **b** Patients with R-based chemoimmunotherapy had superior OS than patients with chemotherapy (120 *vs.* 72 months, *P* < 0.001)

We further analyzed the survival data of these patients. The median follow-up time from the initial treatment was 36 months (range: 2 – 168 months). The median PFS (110 *vs.* 49 months, *P* = 0.001) and OS (120 *vs*. 72 months, *P* < 0.001) time of patients in rituximab group were superior to those of patients without rituximab therapy (Fig. 1a and b).

### CLL group

**Fig. 2:**
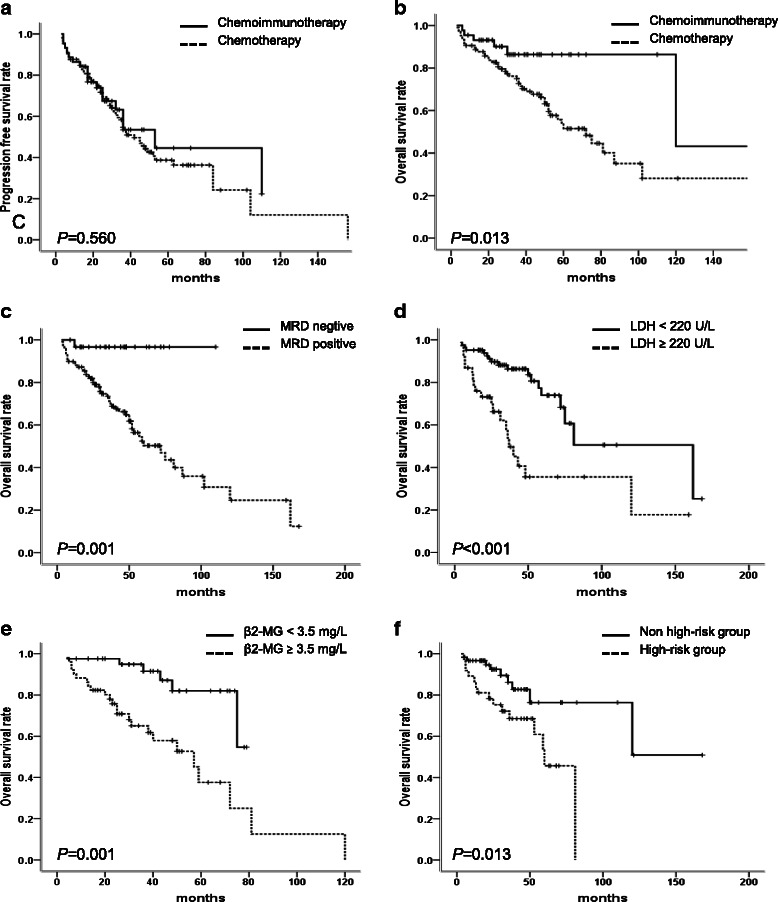
The survival of patients in subgroups. **a** Patients with R-based chemoimmunotherapy had similar PFS as patients with chemotherapy (53 *vs.* 42 months, *P* = 0.560) in CLL group. **b** Patients with R-based chemoimmunotherapy had superior OS than patients with chemotherapy (120 *vs.* 72 months, *P* = 0.013) in CLL group. **c** Patients achieving MRD^−^ had superior OS time than patients with MRD ^+^ in CLL group (not reached *vs.* 72 months, *P* = 0.001). **d** Patients with LDH < 220 U/L had better OS time than patients with LDH > 220 U/L in CLL patients (162 *vs.* 37 months, *P* < 0.001). **e** Patients with β2-MG < 3.5 mg/L had better OS than patients with β2-MG > 3.5 mg/L (not reached *vs.* 57 months, *P* = 0.001). **f** Patients with high-risk cytogenetic abnormality had inferior OS than patients without high-risk cytogenetic abnormality (60 *vs.* not reached, *P* = 0.013)

The median OS time of patients in rituximab group was superior to that of patients in chemotherapy group (120 *vs.* 72 months, *P* = 0.013) (Fig. 2b). However, there was no difference of the median PFS between chemoimmunotherapy and chemotherapy group (53 *vs.* 42 months, *P* = 0.560) (Fig. 2a). Univariate analysis showed that patients with MRD < 1 %, LDH < 220 U/L, obtaining CR or PR, β2-MG < 3.5 mg/L and non high-risk cytogenetic abnormality had superior survival time (Table 6, Fig 2c, d, e and f). However, in multivariate analysis, no independent factor related to PFS and OS was observed. In four CLL patients with no response to rituximab-based treatment, only one CLL patient with del (p53) received allo-PBSCT due to disease progression after three cycles of RFC (Table 3, case 2) and was still alive with the OS of 44 months. Other three patients with complex karyotype or ZAP-70 positive were dead with the survival time of 6, 12 and 30 months, respectively (Table 3).

**Table 6:** The comparison of PFS and OS in the subgroup of CLL patients

Group	Numbers	PFS (range)	*P* value	OS (range)	*P* value
MRD			0.002		0.001
≥ 1 %	117	36 (26.8 ~ 45.1)		72 (63.4 ~ 98.6)	
< 1 %	31	Not reached		Not reached	
LDH			0.001		<0.001
≥ 220U/L	38	29 (19.2 ~ 38.8)		37 (28.5 ~ 45.5)	
< 220U/L	83	52 (35.0 ~ 68.9)		162 (70.7 ~ 253.3)	
CR			<0.001		<0.001
Yes	38	110 (27.2 ~ 192.8)		63 (41 ~ 85)	
No	111	36.0 (29.0 ~ 43.0)		25 (11 ~ 39)	
CR + PR			<0.001		<0.001
Yes	88	63.0 (36.0 ~ 90.0)		120.0 (93.2 ~ 146.8)	
No	61	25.0 (18.1 ~ 31.9)		43.0 (28.8 ~ 57.2)	
β2-MG (mg/L)			0.003		0.001
≥ 3.5	51	32 (22.7 ~ 41.3)		57.0 (38.1 ~ 75.9)	
< 3.5	42	Not reached		Not reached	
Cytogenetic abnormalities			0.005		0.013
High -risk	37	28 (14.5 ~ 41.5)		60 (49.2 ~ 70.8)	
Non high-risk	61	84 (17.8 ~ 150.2)		Not reached	

### FL group

**Fig. 3:**
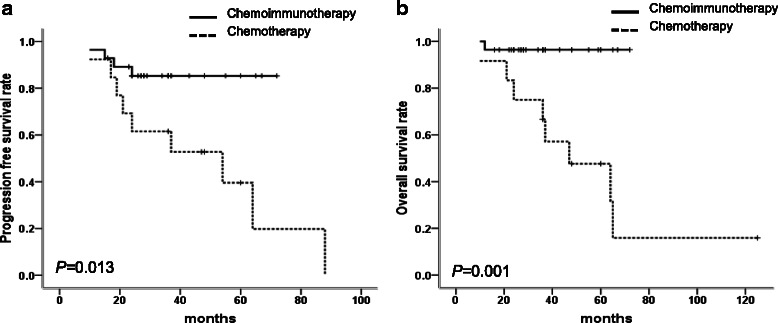
The comparison of outcomes between R-based chemoimmunotherapy and chemotherapy groups in 41 FL patients. **a** Patients with R-based chemoimmunotherapy had superior PFS than patients with chemotherapy (not reached *vs.* 47 months, *P* = 0.013). **b** Patients with R-based chemoimmunotherapy had superior OS than patients with chemotherapy (not reached *vs.* 54 months, *P* = 0.001)

The median PFS in non-R and R groups were 47 months and not reached (*P* = 0.013) and the median OS in non-R and R group were 54 months and not reached (*P* = 0.001) (Fig. 3a and b).

### Other BLPD group

In MZL group, patients with R-based treatment had superior PFS (*P* = 0.034), but no significant difference in OS. Moreover, due to the diversity of clinical features and limited number of patients in other BLPD groups, significant difference of OS time was not available between R and non-R groups in WM/LPL, HCL and BLPD-U patients. Only three HCL patients received rituximab as the initial treatment, the 5-year OS rate was 100 %. However, the 5-year OS rate was 72.7 % in 14 HCL patients who received other treatments such as interferon, fludarabine or chlorambucil.

### Toxicity

The most common side-effect was cytopenia in this study. The rates of grade 3–4 and 1–2 anemia was 31.5 % *vs.*30.0 % and 25.2 % *vs.* 24.1 %, respectively, in R and non-R groups during the courses of treatment. In addition, the rates of grade 3–4 and 1–2 thrombocytopenia were 15.1 % and 16.7 %, and 18.3 % and 26.1 % in R and non-R groups, respectively. The incidence of side-effect about anemia and thrombocytopenia was similar between two groups (*P* > 0.05). However, grade 3–4 neutropenia occurred more often in the chemoimmunotherapy group compared to non-R group (39.4 % *vs.* 17.3 %, *P* < 0.001). Patients were well tolerated to the course of bone marrow suppression when they were administrated with blood cell stimulating factor and infused blood components. The incidence of infection was similar in the two groups (43.0 % *vs.* 41.3 %, *P* > 0.05), and the common sites of infection were lung, gastrointestinal tract, upper respiratory and mouth. Transiently increased level of transaminase but restored after the treatment of liver protection was observed in 8.6 % (11/128) of patients. Twenty-one patients developed chill, fever or skin itch during the infusion of first–dose rituximab and the symptoms disappeared after symptomatic treatment. Two patients discontinued with the rituximab treatment because of severe angioedema and repeated diarrhea. One patient was diagnosed with pancreatic cancer after completion of six courses of the rituximab treatment for two months. Another patient who received four courses of R-CHOP was died of brain tumor with the survival time of 23 months. No treatment-related death was observed.

## Discussion

In the last decade, rituximab-based chemoimmunotherapy has been reported to improve not only ORR and CR rate but also PFS and OS of B-iNHLs patients in Western world [9, 17, 18]. However, due to the low incidence of B-iNHLs in China, there is still lack of detailed data about incidence, genetic abnormalities, prognostic factors of B-iNHLs in Chinese patients. Our previous reports and other studies in China have shown that there could be some discrepant features between Chinese and western patients, such as relatively lower age onset and different prognostic factors in Chinese CLL patients [19, 20]. Moreover, the frequency and mutation status of IgVH gene expression in Chinese CLL patients are significantly different compared with western patients, however, the mechanism is currently unclear [21]. These findings suggest there might be some potential differences including pathogenesis, treatment response and prognosis in Asian CLL patients. To explore the efficacy, safety and prognostic effect in Chinese B-iNHLs patients with rituximab-based chemoimmunotherapy as an initial therapy, we retrospectively analyzed the clinical data of B-iNHLs patients hospitalized in our center since1999.

FCR regimen has now become the standard first-line therapy for CLL. The results from the famous German CLL Study Group (GCLLSG) (CLL8 trial) have confirmed that the addition of rituximab to chemotherapy could significantly improve the outcome of CLL patients with enhanced PFS and OS [9]. The rates of CR (44 % *vs.* 22 %, *P* < 0.0001) and OR (90 % *vs.* 80 %, *P* < 0.0001) were obviously higher in FCR group compared to patients in FC group. Moreover, patients in FCR group had superior PFS (65 % *vs.* 45 %, *P* < 0.0001) and OS (87 % *vs.* 83 %, *P* = 0.01) than patients in FC group. Other than more incidence of grade 3–4 neutropenia and leukocytopenia in FCR group, there are no increase of other side-effects including severe infections. Another study from MD Anderson Cancer Center (MDACC) achieved the similar excellent result [22]. Our results were consistent with previous reports. Patients receiving R-based chemoimmunotherapy had significantly higher rates of OR and CR than patients receiving other therapies. Moreover, R-based treatment can obviously increase the OS time of CLL patients from 72 to 120 months. Similarly, RFC regimen was superior to FC regimen in Chinese CLL patients with higher rates of CR (44.4 % *vs.* 19.4 %, *P* = 0 · 039) and OR (81.5 % *vs.* 51.6 %, *P* = 0.017). However, we did not observe the difference in PFS and OS between the FCR and FC group, and different PFS in CLL patients in R and non-R groups. We thought the reasons might be as follows: the limited cases in RFC and FC group (27 *vs.* 31 cases); most (64.5 %) of patients receiving FC therapy were before 2008, however, most (74.1 %) of patients received RFC therapy after 2008 with relatively shorter follow-up time; in addition, FC could improves PFS but not OS in CLL patients [23].

It is worthy to note, in Chinese CLL patients, we found patients with β2-MG < 3.5 mg/L, LDH < 220 U/L, ZAP-70 negative and with non high-risk genetic abnormality had higher CR rate after receiving R-based treatment. And more patients with rituximab-based treatment achieved MRD negative. Survival analysis also confirmed CLL patients with MRD < 1 %, LDH < 220 U/L, achieving CR or PR, β2-MG <3.5 mg/L and non high-risk cytogenetic abnormality had superior outcome compared to control patients, suggesting CLL patients with β2-MG < 3.5 mg/L, LDH < 220 U/L, ZAP-70 negative, and non high-risk genetic abnormality could be more appropriate candidates for rituximab-based therapy.

Similarly, the combination of rituximab and chemotherapy has been confirmed to improve the outcome of new diagnosed FL patients with superior CR, ORR, PFS and OS in several randomized trials. The combination has now become the standard first-line therapy for FL [18, 24]. In the present study, our results also showed excellent response and outcome in Chinese FL patients who received R-based therapy. Moreover, FL patients with low or medium risk FLIPI score could benefit more from the R-based regimens to achieve higher CR rate.

Rituximab-based regimens have also been recommended as an initial therapy for most patients with WM according to International Workshop on WM consensus [25]. DRC regimen (dexamethasone, rituximab, and cyclophosphamide), a mainly primary choice, was reported to have 35 months of median PFS and 95 months of median OS [25]. However, rituximab alone is not a good choice for LPL/WM patients due to lower response rate and the risk of transiently increased level of IgM, which can lead to hyperviscosity [26]. Whether rituximab alone or combined with chemotherapy should be used as the front-line treatment in MZL or HCL patients is still controversial [27, 28]. Nevertheless, rituximab alone or in combination with chemotherapy is considered as first-line therapy in MZL patients who are not fit for surgery or splenectomy [5]. Similarly, rituximab is currently used in the patients with purine analog relapse and resistance as purine nucleoside analog pentostatin and cladribine have shown promising activity in untreated HCL patients with 80-90 % of CR rate and near 100 % of ORR, resulting in longer remission duration time compared to patients treated with interferon alpha [29]. In our study, possibly due to the limited number of patients in MZL, LPL, HCL groups and the diversity of clinical features in BLPD-U group, we didn’t find any difference in treatment response, PFS and OS between the R and non R groups except higher CRR in LPL patients and better 5-year PFS in MZL patients received R-based therapies. Whether R-based chemoimmunotherapy could be a better choice for these patients needs further investigation in larger samples.

## Conclusions

To our knowledge, this report assessed the efficacy and safety of rituximab–based chemoimmunotherapy in the largest cohort of Chinese patients with B-cell indolent lymphomas. Our data confirmed that rituximab–based chemoimmunotherapy as the first-line therapy is more efficacious than other treatments in newly diagnosed B-iNHL patients with superior treatment response and prolonged survival time. Moreover, we identified subpopulations could benefit more from the R-based regimens in CLL groups. With the exception of 3–4 neutropenia occurring more often in the chemoimmunotherapy group, other side-effects didn’t increase. Our results strongly support rituximab–based chemoimmunotherapy as an effective and safe treatment option in Chinese B-iNHL patients.

## Abbreviations

B-iNHLs: B cell indolent non-Hodgkin lymphomas
CLL: Chronic lymphocytic leukemia
FL: Follicular lymphoma
NMZL: Nodal marginal zone lymphoma
SMZL: Splenic B-cell marginal zone lymphoma
LPL/WM: Lymphoplasmacytoid lymphoma/Wadenström macroglobulinemia
HCL: Hairy cell leukemia
BLPD-U: Chronic B lymphoproliferative disease undefined
β2-MG: β2-microglobulin
LDH: Lactate dehydrogenase
ZAP-70: Zeta-chain-associated protein kinase 70
MRD: Minimal residual disease
Allo-HSCT: Allogeneic hematopoietic stem-cell transplantation
R: Rituximab
R-FC: Rituximab fludarabine and cyclophosphamide
CR: Complete remission
PR: Partial remission
SD: Stable disease
PD: Progression disease
ORR: Overall response rate
PFS: Progression-free survival
OS: Overall survival
